# Residents' intentions and actions after patient safety education

**DOI:** 10.1186/1472-6963-10-350

**Published:** 2010-12-31

**Authors:** José D Jansma, Cordula Wagner, Arnold B Bijnen

**Affiliations:** 1 Foreest Medical School, Medical Center Alkmaar, Wilhelminalaan 12, 1815JD Alkmaar, the Netherlands; 2 EMGO Institute for Health and Care Research, VU University Medical Center, Van der Boechorststraat 7, 1081BT Amsterdam, the Netherlands; 3 NIVEL Netherlands Institute for Health Services Research, Otterstraat 118 - 124, 3500BN Utrecht, the Netherlands; 4 VU University Medical Center, Institute for Education and Training, Van der Boechorststraat 7, 1081BT Amsterdam, the Netherlands

## Abstract

**Background:**

Medical residents are key figures in delivering care and an important target group for patient safety education. The objective of this study was to assess residents' intentions and actions concerning patient safety improvement after patient safety education.

**Methods:**

Four multi-specialty 2-day patient safety courses were organized, in which residents from five Dutch hospitals participated. At the end of these courses participants were asked to formulate an action point to improve patient safety. Three months later semi-structured interviews were conducted to reveal actions that were taken, factors that had influenced their behaviour and reactions concerning the education. An inductive theory approach was used to analyze transcriptions.

**Results:**

Out of 71 participants, sixty-nine (97%) residents were interviewed. In total they had formulated 91 action points, which mainly focused on: 'Improving organization of own work/Follow policies' and 'Improving culture/Educating colleagues about patient safety'. Sixty-two (90%) residents declared to have taken action, and 50 (55%) action points were fully carried out. Most actions taken were at the level of the individual professional, rather than at the level of their social or organizational context. Results of actions included adjusting the structure of their own work, organizing patient safety education for colleagues, communicating more efficiently and in a more structured way with colleagues, and reporting incidents. Promoters for action included: 'Awareness of the importance of the action to be taken', 'Supportive attitude of colleagues' and 'Having received patient safety education'. Barriers included: 'Impeding attitude of colleagues', 'High work-pressure', 'Hierarchy' and 'Switching of work stations'.

**Conclusions:**

After patient safety training, residents reported various intentions to contribute to patient safety improvement. Numerous actions were taken, but there still is a discrepancy between intentions and actual behaviour. To increase residents' participation in patient safety improvement, educational efforts should be supplemented with actions to remove experienced barriers, most of which are related to the residents' social and organizational context.

## Background

Global attention to improving patient safety arose after studies had revealed a large extent of harm due to adverse events [1-4]. A major part of these events is believed to be avoidable, which drives the search for improvement interventions [2-5]. Policy plans in many countries have acknowledged the need to incorporate patient safety principles into graduate medical education [6-9]. Since 1999, the United States Accreditation Council on Graduate Medical Education (ACGME) requires residents to demonstrate competence in 'practice-based learning and improvement' and 'systems-based practice', which has resulted in multiple patient safety educational interventions [10-13].

In the Netherlands, patient safety is also appointed as one of the topics that needs to be addressed in multi-specialty education for residents [14]. Patient safety improvement often involves non-technical skills and therefore patient safety education is not necessarily discipline-specific. Examples of patient safety topics include the improvement of information transfer among health care workers and adverse event reporting and analysis [15,16]. In general, patient safety education aims to create awareness of risks and to induce changes in behaviour to deliver safer care.

However, little is known about the effects of patient safety education on the actual behaviour of residents. Only a few articles [10,17,18] briefly described some intentions and actions resulting from patient safety education for residents. These actions were mostly related to systems redesign, i.e. improvement of information technology, development of new educational programmes and modifications of medical documentation. However, it is not clear whether the residents who were trained had actually undertaken these actions themselves, as the actions could have been assigned to and carried out by other health care workers in the organization [10]. Besides, researchers discovered that 40-50% of the recommended actions had been abandoned after the training or had been implemented only partially, but they did not investigate or describe the hindering factors involved [10,17,18].

Our multi-centre study tried to fill these gaps by investigating residents' intentions and actions to improve patient safety after they received patient safety education. This approach is based on the Theory of Planned Behaviour (TPB), which states that intention is the immediate antecedent of behavioural change [19]. We also assessed which factors were perceived by residents as facilitating or hindering the intended actions, and we elicited their personal evaluation of the patient safety course. An in-depth understanding of the barriers to change and promoters for achieving change in day to day practice is needed to bridge the gap between best practice and actual clinical care [20], and this understanding can be valuable for the improvement of medical education as well as for the development of health care policies.

## Methods

### Context

Residents from five Dutch hospitals participated in four multi-specialty patient safety courses. The first two courses, which were organized at a large Dutch general teaching hospital, were attended by residents as well as by medical graduates who were not in training to become specialists. In this article, therefore, the term 'residents' refers to both groups of medical graduates.

In total, attending the course involved about 16 hours with plenary sessions and small group sessions. The curriculum was delivered by external speakers, as well as by employees of the hospitals. Among the speakers were physicians, psychologists, legal experts and a sociologist with many years of experience in the field of patient safety research and medical education. The courses differed in the number and the length of the educational sessions. The first two courses were organized in 2007/2008 and consisted of one day, followed in 4 to 6 week intervals by two half days. The third and the fourth course were organized in 2008 and consisted of two full days with an interval of 8 weeks with. The first two and the last two courses somewhat differed in format because the organizational possibilities differed between the settings.

All 4 courses were comparable regarding content and teaching methods and aimed at increasing residents' knowledge, attitudes and skills to recognize and cope with unintended events and unsafe situations in an early stage. The main course topics were: 1] principles of patient safety; 2] human factors; 3] effective teamwork (i.e. communication); 4] contribution to safer care; and 5] medico-legal aspects of patient safety. The learning goals per theme are displayed in table 1. At the end of the course all participants were asked to formulate one action point to improve patient safety.

**Table 1:** Patient safety course themes and learning goals

Themes	**Learning goals: after the course residents can..**.
1. Principles of patient safety	- Explain definitions and give examples of incidents. - Point out main research outcomes concerning the occurrence of adverse events. - Describe the roles of different parties regarding patient safety.
2. Human factors	- Explain how incidents can occur. - Describe the role of human factors with regard to patient safety. - Demonstrate ways to minimize risks related to human factors. - Explain what health care can learn from other high-risk industries.
3. Effective teamwork	- Describe different aspects of effective teamwork. - Recognize aspects of teamwork that can be risky and explain why they can be risky. - Clarify why an open culture is important for improving patient safety. - Explain ways to cope with the hierarchical structure in a hospital while protecting the safety for patients. - Express ways to improve information transfer (verbal and written). - Explain why giving and receiving feedback is important. - Describe how (other) team factors can be improved.
4. Contribution to safer care	- Clarify risks of the hospitals' processes. - Recognize unsafe situations or processes in an early stage. - Demonstrate what should be done when an unsafe situation or process is noticed. - Describe what and how we can learn from analyzing incidents. - Point out different methods that can be used to analyze incidents/risky processes - Demonstrate analysis of own perceived incidents. - Describe other methods that can improve the safety of patients.
5. Medico-legal aspects of patient safety	- Explain the role of protocols. - Demonstrate what to do after an incident has occurred. - Clarify what a patient may be told when an incident has occurred. - Point out how patients want to be approached after an incident. - Describe potential consequences of an incident. - Suggest actions that reduce the risk for claims.

The Scientific Committee of the VU University Medical Center, the Netherlands, provided a waiver for this study. National rules and regulations for health services research were followed.

### Data collection

Three months after the course had ended, participants were approached to participate in semi-structured interviews. We chose this method because we wanted to gain insight into their personal experiences after attending the patient safety course. Time and venue were chosen to suit the preferences of the residents. The duration of the interviews varied from 20 to 90 minutes. In these interviews we asked whether residents had carried out their action points and to what extent. We also asked which barriers and promoters had played a role, what additional actions they had undertaken regarding patient safety and whether the respondent still had intentions to improve patient safety.

All interviews were conducted by the same researcher (JDJ). A question schedule (table 2) was used, but the interviewer was free to follow up on themes that emerged during the interviews. At the start of each interview the interviewer explained that all data were to be processed confidentially and that documents would be coded to ensure anonymity. During the interviews the interviewer made brief short notes of the respondents' answers. Shortly after each interview, these notes were computed in Microsoft Word to create a transcript. Transcripts were mailed to the respondents within three days after the interview. Residents were asked whether the transcript gave an accurate representation of the interview and were given the opportunity to make revisions (member checking).

**Table 2:** Interviews' question schedule

1.	Did you carry out your action point?
	
2.	If yes, what were the results of your actions?/If not, please explain.
	
3.	Which promoters encouraged you to carry out your action point?
	
4.	Which barriers discouraged you from carrying out your action point?
	
5.	Which additional patient safety related actions did you carry out?
	
6.	Do you have patient safety related action points to work on for the future?
	
7.	If you do, which actions did you choose?/If not, please explain.
	
8.	How do you look back on the content and structure of the patient safety course?

### Data analysis

The interview transcripts were entered into qualitative data analysis software Atlas.ti 5.2. An inductive theory approach was used, as our aim was to explore and to explain the process of residents' behaviour. Short descriptors were assigned to text fragments (coding). The first five interviews were open-coded independently by two researchers (JDJ&KM) and the coding system was compared and discussed until full agreement was reached. In line with the agreed system, one researcher (JDJ) coded all the other interviews. Consequently the number of codes was reduced by merging codes that had comparable meanings and axial coding was performed to identify overarching themes and to explore relations. This resulted in a number of promoters for action and barriers to action.

At this stage we searched the literature for theories that could be relevant for further analysis of our data. The levels of health care as defined by Grol and Wensing [20] were used as a basis for further classification. Based on the question "What principal aspects had changed/had to change?", the action points and the corresponding results were classified into three levels: 1] individual professional (related to the resident, i.e. awareness, knowledge, attitude, motivation to change or behavioural routines); 2] social context (related to the residents' department or colleagues, i.e. opinion of colleagues, culture of the network, collaboration or leadership); and 3] organizational context (related to the organization the resident works for, i.e. organisation of care processes, staff, capacities, resources or structures). Promoters for action and barriers to action were also classified into these three levels. The extent of action taken was categorized as well: 1] fully carried out; 2] partly carried out; and 3] not carried out. An action was classified as partly carried out if the resident had taken action but had not yet reached their goal, or if not all the required actions had been taken so far.

Any difficulties and uncertainties during the analyzing process were discussed until agreement was reached. Residents' characteristics, the level of the action point and the extent of actions taken were processed in SPSS 15.0. Crosstabs were used to assess if there were correlations between residents' characteristics and the level of their action points or the extent of actions taken. We used a probability of *p *≤ .05 (two-tailed). Only in case of notable correlations this is mentioned in the results section.

## Results

In total, 69 (97%) of 71 residents were interviewed. Twenty-six (38%) of them had attended the patient safety course at the academic centre. The participants worked in five different hospitals, and specialized in eighteen different disciplines. Most of them worked in general surgery (n = 12, 17%), anaesthesia (n = 11, 16%) or paediatrics (n = 10, 15%). Member checking resulted in minor transcript revisions and adjustments by 13 (19%) residents. Respondents' characteristics are shown in table 3.

**Table 3:** Characteristics of respondents (n = 69)

Age - years	
Range	24.8-43.3
Median age	29.7
	
Sex - n (%)	
Male	26 (38)
Female	43 (62)
	
Discipline - n (%)	
Surgical	33 (48)
Non-surgical	36 (52)
	
Residency training - n (%)	
Yes	56 (81)
No	13 (19)

### Action points

In total, 91 action points were formulated by 68 residents (mean per resident: 1.35). One resident formulated no action point and was therefore excluded from the analysis. Although we asked residents to formulate one action point, 17 (25%) residents formulated two action points and 3 (4%) formulated three action points. Intended actions most often aimed at changing the social context or the individual professional. Less often their intentions aimed at changing the organizational context (table 4).

**Table 4:** Action points and results of actions

Level: Who needs to change? n (% of total)	**Action point**, n (% of total)	Extent of taken actions, n (% within action point)	Example of declared results
Individual professional, 42 (46%)	Improving organization of own work/Follow policies, 18 (20%)	14 (78%) fully	*I adjusted my own workstation and set of tasks in order to improve patient safety*.
		1 (6%) partly	*I tried to check the medication use of patients more frequently, but I am not doing it as often as I intended*.
		3 (17%) not	
	
	Improving own information transfer towards colleagues, 9 (10%)	7 (78%) fully	*When I need to consult my supervisor, I always try to use the model for structured information transfer that was explained at the patient safety course*.
		1 (11%) partly	*I did communicate in a more structured way with my colleagues, but I did not use the model that was explained during the course, although I intended to do sot*.
		1 (11%) not	
	
	Learning from mistakes/Reporting incidents, 9 (10%)	3 (33%) fully	*I reported incidents I was involved in*.
		2 (22%) partly	*I did signal some incidents, but I did not report them because they were related to nurses' tasks*
		4 (44%) not	
	
	Improving writing in patient records, 4 (4%)	4 (100%) fully	*I avoid the use of abbreviations when I am writing in patient records and when I see that colleagues have used them I often spell them out for them*.
	
	Improving communication with patients, 1 (1%)	1 (100%) fully	*Now I am always checking if the patient fully understands the information that was provided about the upcoming procedures*.

Social context, 43 (47%)	Improving culture/Educating colleagues about patient safety, 17 (19%)	14 (82%) fully	*I invited one of the speakers of the patient safety course to speak about patient safety at our department*.
		1 (6%) partly	*I did approach colleagues about a patient safety issue but I don't believe that it has changed anything in their behaviour*.
		2 (12%) not	
	
	Improving communication within the health care team, 15 (16%)	4 (27%) fully	*I explained the model for structured information transfer to some novice nurses of my department*.
		4 (27%) partly	*I made a plan to introduce the model for structured information transfer at my department, but this has not been carried out yet*.
		7 (47%) not	
	
	Improving protocols/policies, 11 (12%)	2 (18%) fully	*I wanted to know who is responsible for filling the departments' medication wagon, now I know and I if necessary I can approach this person directly*.
		6 (55%) partly	*I have selected an article about the EWS for an upcoming presentation for my colleagues*.
		3 (27%) not	

Organizational context, 7 (8%)	Improving hospital's digitalization, 5 (5%)	2 (40%) partly	*I contacted the pharmacists to discuss the possibilities for simplifying the medication prescription system. It is still on their agenda*.
		3 (60%) not	
	
	Advocate for better/new equipment, 2 (2%)	2 (100%) partly	*I presented the need for new equipment to the person in charge, but as far as I know no changes have been made so far*.

EWS = Early Warning Score. OR = Operating Room. TOP = Time Out Procedure.

*Partly indicates that the resident did take action, but their goal was not (yet) reached, or not all required actions have been taken.

### Results of actions

Sixty-two (90%) residents declared to have taken action on the action point they had formulated, although just 50 (55%) action points were fully carried out. Results of actions included: adjustment of own structure of work; organizing patient safety education for colleagues; more efficient and structured communication with colleagues; and reporting of incidents. Action points on the level of the individual professional were most often carried out fully (n = 29, 69%), followed by actions point on the level of the social context (n = 20, 47%). On the level of the organizational context, none of the intended action points were fully carried out. A selection of the reported results are shown in table 4.

Sixty (87%) residents mentioned additional actions they had taken to improve patient safety, apart from their original action point(s). These additional actions were related to the individual professional (n = 65, 76%) or to the social context (n = 21, 24%). The original action points chosen within these levels (table 4) were all found as well in the additional actions that the residents declared to have taken. Moreover, the additional actions on the level of the individual professional led to additional results: 1] an increased alertness in daily practice/awareness of the importance of patient safety, and 2] improved knowledge about patient safety, due to reading the literature provided during the patient safety course.

### Promoters for action

In total, promoters for carrying out the action points were mentioned 43 times (mean per resident: 0.63). These were mainly related to the individual professional and their social context. Promoters in relation to the individual professional mostly were, 'Awareness of the importance/usefulness of the action to be taken' and 'Having received patient safety education'. This is illustrated by the following two interview quotations: "*The model for structured information transfer is very useful for me. If I hesitate to contact my supervisor, this structure helps me to organize and strengthen my questions, which enlarges my self-confidence and improves the communication with my supervisor*" and "*The patient safety course made me aware of the extent and the nature of the errors that occur in health care. This made me realize how much additional value a novice computer system could have for patient safety*".

Promoters related to social context mostly were a 'Supportive attitude of colleagues' and an 'Open culture'. This is illustrated by the following two quotations: "*When I contacted the pharmacists to discuss the possibilities for simplifying the medication prescription system, they directly acknowledged the importance of the issue and put it on their agenda. Their supportive reaction stimulates me to contact them again if I notice another medication safety problem*" and "*At my department there is an open culture in which physicians openly talk about their errors and complications, this encourages me to also discuss patient safety issues I experience in my work*".

Organizational context promoters were mentioned to a lesser extent, i.e. "*In the hospital I work, attention was paid regularly to patient safety improvement. This stimulated me to also be more alert for potential slips*" and "*A visitation is planned on my department, which stimulates us to improve the organization of our department. I believe this contributes to improving patient safety*". An overview of the promoters mentioned is given in table 5.

**Table 5:** Promoters for carrying out action points

Level, n (%)	Promoter	Mentioned, n (%)
Individual professional, 19 (44%)	Awareness of the importance/usefulness of the action to be taken	10 (23%)
	Having received patient safety education	4 (9%)
	Interested in subject/Motivated to change	2 (5%)
	More experience gives opportunities to focus on other aspects of work	2 (5%)
	Being former member of reporting committee	1 (2%)
		
Social context, 18 (42%)	Supportive attitude of colleagues	10 (23%)
	Open culture	5 (12%)
	Other residents from department also attended the patient safety course	2 (5%)
	Reaction received after incident report	1 (2%)
		
Organizational context, 6 (14%)	Increased attention to patient safety	3 (7%)
	Upcoming visitation	1 (2%)
	Reporting incidents does not take much time	1 (2%)
	New work station stimulates exploring the environment	1 (2%)

### Barriers to action

Barriers that hindered residents in carrying out the action points were mentioned more than twice as frequently as promoters, 98 times (mean per resident: 1.44) in total. These barriers were mainly related to the organizational context (n = 40, 43%) and the social context (n = 33, 35%).

Barriers mentioned most frequently in relation to the organizational context were 'High work-pressure' and 'Switching of work stations'. This is illustrated by the following two interview quotations: "*On my department there are insufficient residents to fulfil all the required tasks, we all are experiencing a very high work-pressure and I don't have time to pay extra attention to safety issues*" and *"This is such a huge organization, almost all intentions for changes silt up in bureaucracy"*. The organizational context barrier 'Hugeness of organization/Bureaucracy' was of more concern in the academic setting. The barrier 'High work-pressure', on the other hand, was mostly mentioned in relation with a non-academic context, i.e. *"I experience a much higher work-pressure in the non-academic teaching hospital. In academic centres we more often find uncommon clinical pictures and we are getting more time per patient to be able to immerse ourselves in the best evidence literature"*.

Barriers related to the social context mostly concerned 'Impeding attitude of colleagues' and 'Hierarchy'. This is illustrated by the following two interview quotations: "*The need to change is not felt by my colleagues*" and "*Our supervisors do not listen to our input concerning the improvement of patient safety*".

A smaller number of barriers mentioned were related to the individual professional (n = 21, 22%), i.e. "*I forgot what I had chosen as my action point*", "*My motivation for taking this action just diminished*" and *"I don't want to initiate these changes for my department because in my opinion this rather is a task for our supervisors"*. An overview of the barriers mentioned is given in table 6.

**Table 6:** Barriers to carrying out action points

Level, n (%)	Barrier	Mentioned, n (%)
Individual Professional, 25 (26%)	Doubts about usefulness/Loss of motivation	6 (6%)
	Action point forgotten	5 (5%)
	Don't want to be initiator for changes	5 (5%)
	Experienced no problems anymore	4 (4%)
	Hard to break through routines/People are fallible	4 (4%)
	Formulation of action point was demanded	1 (1%)
		
Social context, 33 (35%)	Impeding attitude colleagues	16 (16%)
	Hierarchy/Dependency on supervisors	10 (10%)
	Poor communication with colleagues	4 (4%)
	Poor accessibility of colleagues	3 (3%)
		
Organizational Context, 40 (43%)	High work-pressure	18 (18%)
	Switching of work stations	10 (10%)
	Hugeness of organization/Bureaucracy	7 (7%)
	ICT problems/Limited user-friendliness of reporting system	3 (3%)
	Residents' short working period at a department	2 (2%)

### Future action points

Fifty-four (78%) residents declared to have plans for patient safety improvement. Twenty of them said they wanted to continue carrying out their previously selected action point(s). The intentions that still remained were mainly related to incident reporting, improving communication within the health care teams, educating colleagues and improving the organization of their own work. Reasons for not having any further plans for taking action included upcoming switch of work stations, high work-pressure or upcoming maternity leave.

### Reactions concerning educational program

Most residents were very enthusiastic about the course, they mentioned it had been enjoyable and interesting, and they acknowledged the importance and usefulness of teaching these issues to residents (mainly positive, n = 46, 67%; positive and negative aspects mentioned, n = 22, 32%; mainly negative, n = 1, 1%).

The multi-disciplinary approach was praised by most residents, e.g. "*I found it very useful to attend this course together with residents from other disciplines, it creates opportunities to discuss and learn about strategies which other disciplines use for coping with problems concerning patient safety*". However, some residents valued the gathering of multiple specialties in a negative way: "*In my opinion it was a pity that the examples that came up during the course were often derived from other disciplines and were therefore not relevant for the specialty I am working for"*.

Residents shared the opinion that the course could best be given in the first period of residency training, as beginning residents are often searching for good work strategies and are therefore best accessible: "*I almost finished my residency training and therefore the course material was not very new to me, as I had taught myself how to cope with unsafe situations over the years. I think it would be best to attend this course at some point during the first two years of residency training*".

Several participants mentioned that the information learnt and the action points slowly faded away after the course, and that therefore it would be helpful to organize refresher moments once in a while. Even the interview itself was often evaluated as very useful, as it was considered a good opportunity to consider the patient safety principles again. As a remedy against forgetting the action point it was suggested to send reminders with action point descriptions to course participants. Residents often mentioned that they had found it quite difficult to put the course content into practice and to carry out the intended actions once they were back at the hospital. Many residents stated that it would have been easier to take action if more of their colleagues also had attended the patient safety training, referring not only to their fellow residents but to nurses and supervisors as well.

## Discussion

With the increasing attention to providing patient safety education to residents, it is important to gain insight in the effectiveness of those initiatives and factors contributing to their success. Interviews with 69 residents from five different hospitals revealed that the residents very positively received our patient safety course. After attending the course they were motivated to improve patient safety and to some extent were able to carry out their formulated action points for patient safety improvement. These action points were closely related to the themes that emerged during the patient safety course and focused on changing their own behaviour or their context. The actions intended most frequently, as well as the ones carried out most frequently were: 'Improve organization of own work/Follow policies' and 'Improve culture/Educate colleagues about patient safety'. Intentions related to the participants themselves were carried out more often than intentions on the departmental or organizational level. These latter two levels were also most often involved in the barriers that residents experienced in taking action, mostly 'High work-pressure', 'Impeding attitude colleagues', 'Hierarchy' and 'Switching of work stations'. Promoters for action included 'Awareness of the importance of the action to be taken', 'Supportive attitude of colleagues' and 'Having received patient safety education'. These findings are in line with previous studies that gave insight into the factors inhibiting incident reporting by doctors [21,22].

For several reasons residents are important key-figures with a unique view on risky aspects of health care [23], which makes them particularly suited for defining system change recommendations [10,17]. Firstly, residents provide much of the direct patient care [24]. Secondly, residents have a broader perspective on health care than most health care workers as they switch work stations frequently. By working in various settings residents can easily experience what the differences are and what are the best practices. However, residents are often not in the best position to carry out system changes. For example, as residents are often working at a hospital/department for only a short period, it is tougher for them to accomplish improvement interventions that require continuous efforts. Besides, the fact that residents switch work stations regularly has a negative influence on the feeling of involvement and simultaneously on the urge to improve their context. Moreover, we found that the dependency on colleagues could prevent them from taking actions, for example if supervisors are not encouraging residents to offer suggestions or if they are hindering patient safety improvement.

The TPB states that attitude, subjective norm and behavioural control are the factors that influence one's intentions and thus indirectly one's behaviour [19]. The barriers that we found might be weighed in relation to these factors, which might give directions for improvements. For our residents, attitude was not a major inhibiting factor, as the residents were free to choose their own action point. However, barriers related to subjective norms and perceived behavioural control were mentioned frequently, like a discouraging attitude of colleagues, in particular of residents' supervisors, and high work-pressure. Barriers fitting these two factors of the TPB were predominantly related to the social and organizational context of the residents.

These barriers are hard to overcome by educating residents, but they could be tackled by training the context of the residents as well and by adjusting policies to stimulate the creation of so-called a generative culture, in which there is active participation at all levels and where safety is perceived to be an inherent part of the business [25]. Some other barriers could be tackled more easily. For instance, forgetting one's action point could be prevented by sending residents reminders with descriptions of their action points. Stimulating residents to make a plan for carrying out their improvement actions, e.g. by using the Plan-Do-Study-Act (PDSA) cycle, has been demonstrated to be useful as well [26]. Adjustments in the work environment of the residents should be considered as well. For example the use of PDA's has proved to be useful for reporting incidents [27].

It is important to keep in mind that this study was based entirely on declarations of residents, which might have provoked a social desirability bias. We tried to overcome this limitation by letting an independent researcher conduct all the interviews and by underlining the confidentiality of our study method. As with all qualitative research, the analysis of the data may be sensitive to interpretation bias. We tried to reduce this bias at several stages of our research process. First, we tried to ensure that we had drawn up a realistic representation of the respondents' view by means of member checking. Later on, during the coding process, other researchers were involved in the analysis as well to prevent interpretation bias.

For future research it would be interesting to further investigate the results of the residents' actions by means of more objective outcome measures, i.e. independent observations in practice, investigations of patient records or data of the hospitals' reporting systems. Additionally, we recommend to further explore and test the possibilities to overcome the perceived barriers.

## Conclusion

In a variety of ways residents had intentions to contribute to patient safety improvement after they attended the patient safety curriculum. Although various actions were carried out, a gap remained between intentions and actual behaviour. Barriers inhibiting patient safety improvement actions by residents mostly are related to the residents' social and organizational context, i.e. High work-pressure', 'Impeding attitude colleagues', 'Hierarchy' and 'Switching of work stations'. Removing barriers within the training context of residents is important for graduate medical educational efforts to increase residents' participation in patient safety improvement.

## Competing interests

The authors declare that they have no competing interests.

## Authors' contributions

JDJ, CW and ABB designed the study, analyzed the data and wrote the manuscript. JDJ collected the data. All authors read and approved the final manuscript.

## Authors' information

José D. Jansma, MSc, is a PhD student of the research programme Patient Safety in the Netherlands. She is working for the Foreest Medical School of the Medical Center Alkmaar, the Netherlands, and the Department of Public and Occupational Health, EMGO Institute, VU University Medical Center, the Netherlands.

Cordula Wagner, MA, PhD, is head of research area 'Quality and Organisation of hospital- and long-term care' at NIVEL Netherlands Institute for Health Services Research. In addition, she is supervisor of the Dutch research programme on patient safety in hospitals at the department of public and occupational health/EMGO Institute, VU Medical Center, the Netherlands.

Arnold B. Bijnen, MD, PhD, is a general gastro-intestinal surgeon at the Medical Center Alkmaar. He has been appointed professor of surgery at the VU University Medical Center for teaching and postgraduate training.

## Pre-publication history

The pre-publication history for this paper can be accessed here:

http://www.biomedcentral.com/1472-6963/10/350/prepub
